# Enhancing Load-Bearing Capacity of Calcareous Sands through Gel Stabilization: A Mechanical and Material Characterization Study

**DOI:** 10.3390/gels10060373

**Published:** 2024-05-28

**Authors:** Jianxiao Gu, Haibo Lyu, Bo Li, Yong Wang, Hui Chen, Xinyi Gao, Xiaojiang Xu

**Affiliations:** 1 Wenzhou Key Laboratory of Intelligent Lifeline Protection and Emergency Technology for Resilient City, College of Architecture and Energy Engineering, Wenzhou University of Technology, Wenzhou 325035, China; 102015295@glut.edu.cn (J.G.); autumnlibo@gmail.com (B.L.); chenhui0306@wzu.edu.cn (H.C.); 17826901201@163.com (X.G.); 2 College of Architecture and Electrical Engineering, Hezhou University, Hezhou 542899, China; bx2015006@glut.edu.cn; 3 College of Civil Engineering and Architecture, Guilin University of Technology, Guilin 541000, China

**Keywords:** mechanical properties, calcareous soils, gel material type, gel material content, secant modulus

## Abstract

Calcareous sands often display wide ring grain configurations, high intragranular porosity, a complex structure, and low grain hardness. These attributes typically do not meet the strength criteria necessary to sustain overlying infrastructure in civil engineering applications. This study investigates gel stabilization techniques, blending gel material with calcareous sand at concentrations ranging from 5% to 22%, followed by curing periods of 3 to 28 days to evaluate the load-bearing capacity. Subsequently, an unconfined compressive test is performed to determine the gel material content in stabilized specimens and investigate the influence of gel material types. The gel material-to-sand ratios employed are set at 5%, 10%, and 16% for Portland cement and 13%, 16%, and 22% for gypsum. After that, a triaxial consolidated undrained test is conducted to assess mechanical behavior, pore water pressure, and mechanical properties. The findings reveal increased dilation, stress–strain hardening, and softening post-yield, regardless of gel material type. Principal stress ratios, secant modulus, and cohesion show a positive correlation with maintenance duration and binder content, with implications for improved load-bearing capacity. The study also elucidates the qualitative relationship between secant modulus *E*_50_ and confining pressure.

## 1. Introduction

Calcareous sands display a high void ratio, making them susceptible to easy fragmentation, with a low level of grain hardness [1,2]. These materials display unforeseen geotechnical medium mechanical characteristics arising from their distinct origin and structure [3,4]. Meeting strength requirements proves challenging for coastal infrastructure construction involving structures like roadway elevations and airfield landing strips, withstanding repeated loads from waves, vehicular traffic, pipeline installation activities, and seismic events [5,6]. Gel material treatment has demonstrated its practicality in mitigating potential risks in sand- or soil-rich regions [7,8,9,10]. Notably, after gel material treatment, calcareous sand demonstrates a considerable strength improvement [11,12].

In recent decades, various gel material enhancers (for example, cement, fly ash, magnesium oxide, calcium carbonate, lime, and gypsum) have been employed to improve the mechanical behavior and control the deformation of soil or sand to support overlying infrastructure in civil engineering [13,14,15,16,17,18]. For instance, some papers [17] observed the utilization of gypsum as a cementing agent to augment the strength and stiffness of calcareous sand. Nevertheless, this approach resulted in brittle failure in a gypsum-cemented specimen. Gu et al. [11] utilized Portland cement as a hardening agent for stabilizing coastal sands, leading to a significant enhancement in the q_ucs_ (unconfined compressive strength) of the solidified specimens. Zeng et al. [19] presented a method that combines phosphor-gypsum with cement to improve the unconfined compressive strength of soils with high water content. Furthermore, to achieve further enhancement in strength, current widely accepted methodologies suggest increasing the content of Portland cement and the integration of supplementary cementitious materials rather than adjusting the gypsum dosage, despite the higher cost and negative effects on resource conservation and economics [20]. Due to their ease of use and cost effectiveness, the gel materials of Portland cement and gypsum are widely employed for reinforcing soft soil or sand. In this study, Portland cement and gypsum were applied to enhance the robustness of calcareous sand, and the mechanical behavior of the stabilized samples was investigated.

Furthermore, thorough research has examined the impact of factors such as gel material content, gel material type, curing duration, density, and loading conditions on strength. While this research has shed light on the mechanical behavior of stabilized specimens in consideration of these variables, it has not been comprehensive in its approach [21,22,23,24,25]. As an illustration, Zhao et al. [26] observed a prolonged hydration reaction in Handan clay, indicating its strength dependence on time. Similarly, Wang et al. [27] reported a corresponding rise in strength concerning higher gel material content in treated specimens. However, there has been limited research in the last decade concerning the impact of gel material type, specimens’ densities, and gel material content. Particularly in the case of calcareous sands, as demonstrated, they showcase a broader spectrum of particle forms, more notable within-grain porosity, a more complex arrangement, and reduced grain hardness. Considering the widespread use of gel materials (Portland cement and gypsum) as construction bonding materials, understanding the mechanical properties and behavior of stabilized specimens in calcareous sand regions is crucial for engineering purposes. As is well established, the strength of stabilized specimens is significantly affected by the gel material type and gel material content, resulting in enhanced bond strength. Therefore, examining these factors is vital for assessing bond strength. This study examines the mechanical performance and strength characteristics of Portland cement-treated and gypsum-treated specimens under different curing periods and confining pressures in triaxial tests.

Various experimental techniques have thoroughly investigated the shear strength of soil treated with gel materials. Notably, among traditional laboratory methods, consolidation triaxial and UCS tests are prominent for assessing the shear strength of soil or sand. Laboratory experiments have demonstrated that the q_ucs_ of stabilized soil increases as the gel material content rose across various soil types, such as Bangkok soft clay [28,29], marine clays [8,30], and silica sand [31]. Moreover, multiple researchers have conducted consolidation undrained triaxial (CU) tests to assess the shear strength of cement-treated soils. These studies revealed that shear strength increases with rising confining pressures and more extended curing periods, as seen in Kasama et al. [32] and Suzuki et al. [33]. Interestingly, soils treated with gel material exhibited notably increased brittleness compared to untreated soils in triaxial and UCS tests [34]. Alongside conventional cement, the introduction of rice husk ash into the soil–cement mixture has been used to improve the cohesion and friction angle of the treated soil [35]. Mahawish et al. [36] conducted scanning electron microscope tests on bonding products with heavy and light biochemical treatments, revealing varied quantities of calcium carbonate crystals in different specimens and offering empirical evidence for the treatment’s efficacy. Additionally, Sharaky et al. [37] and Gu et al. [11] utilized scanning electron microscope tests to study the microstructure of stabilized calcareous sand and investigate the underlying mechanisms of strength enhancement. Despite extensive investigations into the strength enhancement of stabilized soils under different levels of gel material content, curing periods, and confining pressures, a comprehensive understanding of shear strength and mechanical behavior remains absent across different gel material-treated specimens.

This study delves into the mechanical behavior of stabilized specimens, investigating the impact of gel material type on their properties via unconfining compressive strength (UCS) tests. Triaxial undrained (CU) tests explore how the mechanical behavior of stabilized specimens depends on gel material type, confining pressure, density, and gel material content. Initially, CU tests are carried out to evaluate the mechanical properties of stabilized specimens, assessing the influence of confining pressure, gel material content, gel material type, and density on their response. The study examines post-yield strain-hardening and strain-softening in the test results of stabilized specimens. Subsequently, the results of CU tests analyze the mechanical properties of stabilized and loose calcareous sand specimens, including deviatoric stress, friction angle, and deviatoric stress ratio. Finally, an empirical equation, correlating secant modulus *E*_50_ with confining pressure, aids in determining *E*_50_ values for various stabilized specimens, offering guidance for designing infrastructure on calcareous sand regions. The objective of this study is to enhance the understanding of strength evolution for road foundations and transportation engineering facilities. Its insights contribute to the development of more efficient methods for managing and enhancing calcareous sand in geotechnical engineering practices.

## 2. CU Tests Results and Discussion

### 2.1. Impact of Confining Pressure on Mechanical Behavior Response

**Figure 1:**
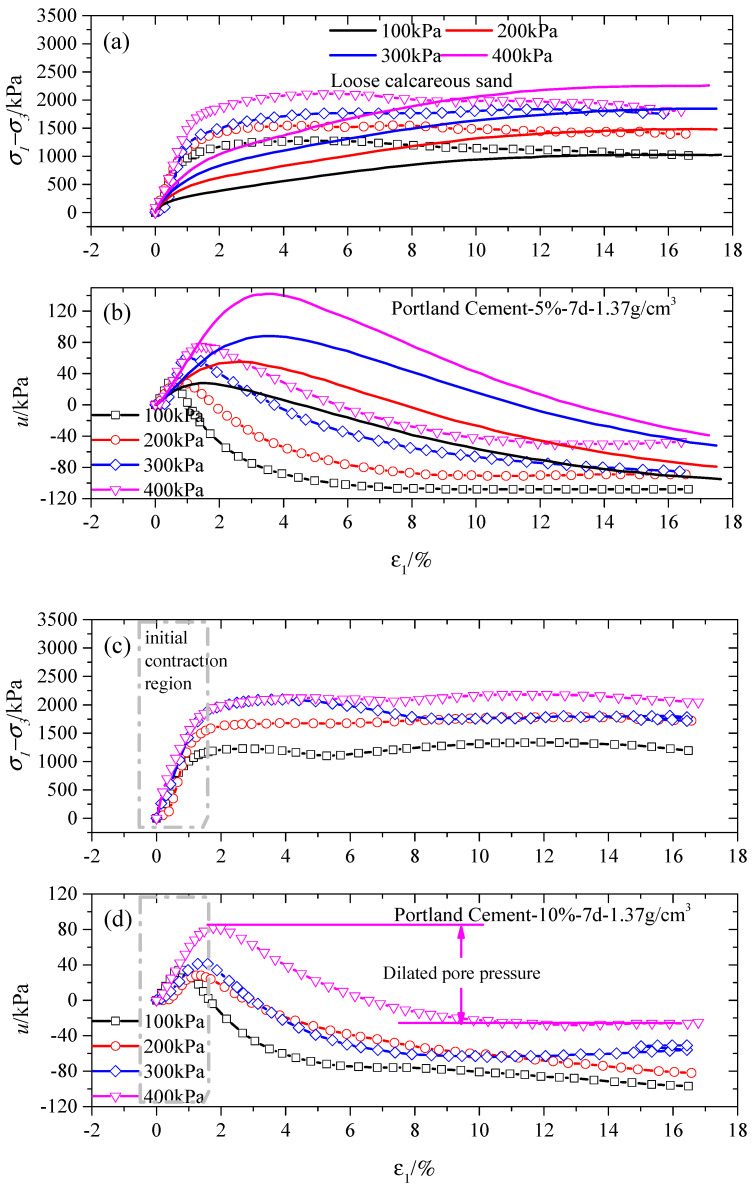

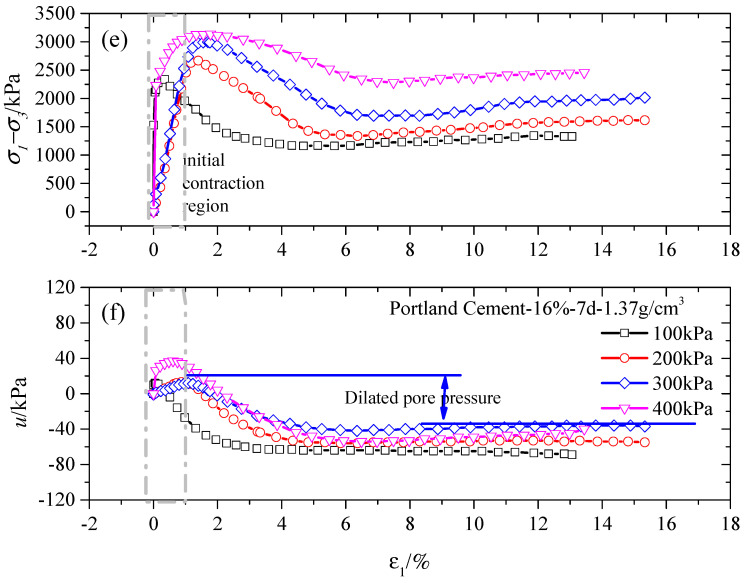
Results for specimens treated with 5%, 10%, and 16% Portland cement content at different levels of confining pressure in the CU tests. (**a**,**c**,**e**) are deviatoric stress versus axial strain; (**b**,**d**,**f**) are pore water pressure versus axial strain.

**Figure 2:**
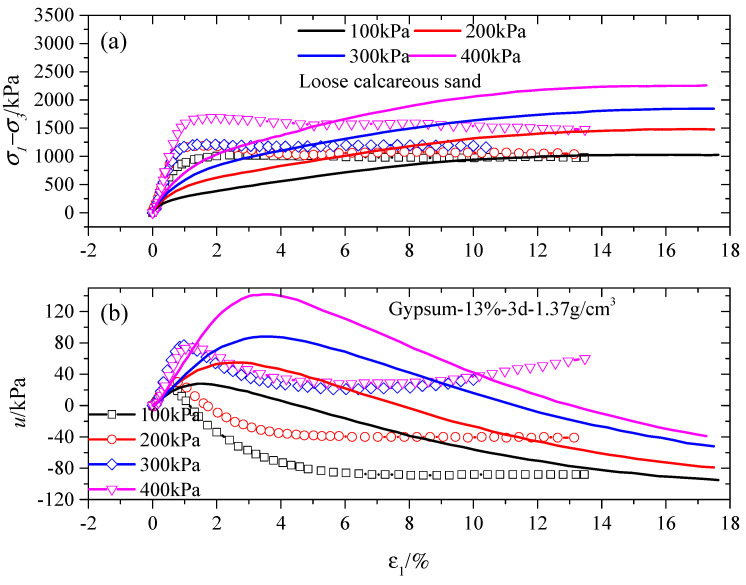

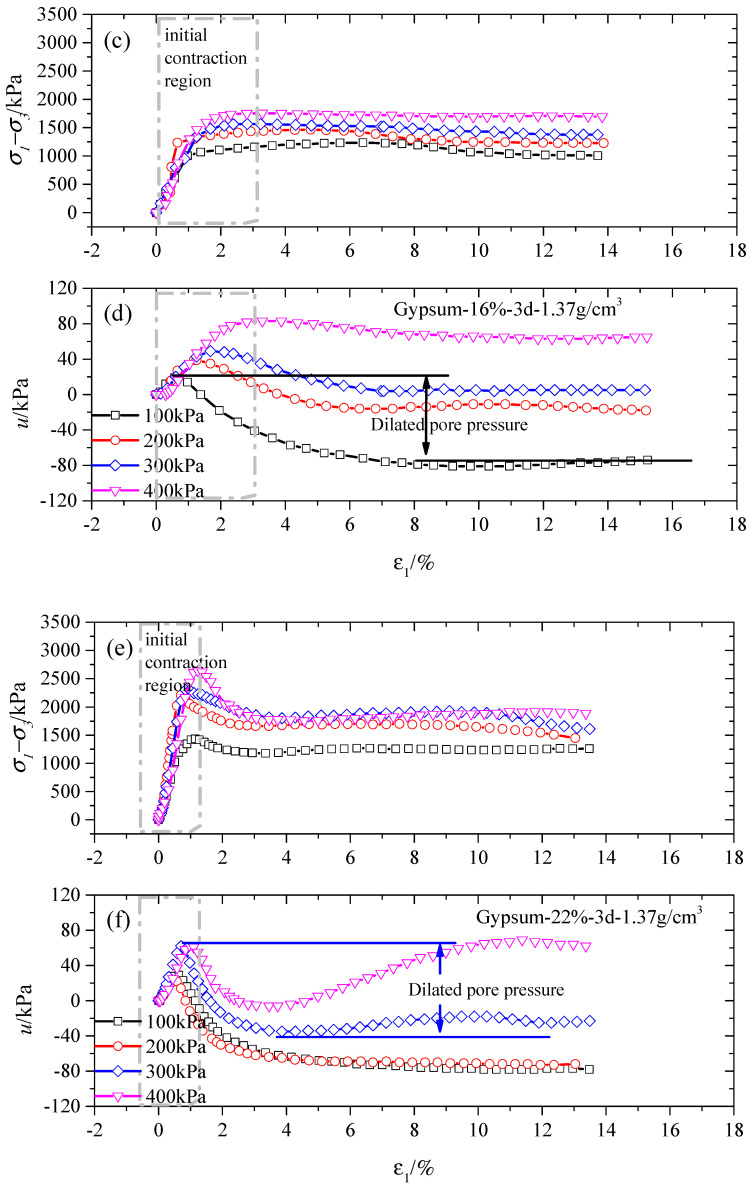
Results for specimens treated with 13%, 16%, and 22% gypsum content at different levels of confining pressure in the CU tests. (**a**,**c**,**e**) are deviatoric stress versus axial strain; (**b**,**d**,**f**) are pore water pressure versus axial strain.

Figure 1 and Figure 2 depict the CU test results for stabilized specimens under various confining pressures with a 7-day curing period for P-stabilized specimens and a 3-day curing period for G-stabilized specimens. It was observed that the curves consistently show a constant slope value at an initial axial strain of 1%, regardless of the gel material content. Additionally, a noteworthy observation was that the peak deviatoric stress ${\left(\sigma_{1}-\sigma_{3}\right)}_{\max}$ experienced a significant escalation as the confining pressure $\sigma_{3}$ increased. To illustrate, a comparison of the peak deviatoric stresses is provided for different Portland gel material contents (5%, 10%, and 16%) under confining pressures of 100 kPa and 400 kPa in Figure 1a, Figure 1c, and Figure 1e, respectively, revealing increases of 65.60%, 62.37%, and 34.32%, respectively. Similarly, for G-stabilized specimens, the ${\left(\sigma_{1}-\sigma_{3}\right)}_{\max}$ under confining pressures of 100 kPa and 400 kPa for different gypsum contents (13%, 16%, and 22%) is displayed in Figure 2a, Figure 2c, and Figure 2e, respectively, showing increases of 66.14%, 41.91%, and 84.04%. Furthermore, the rise in peak deviatoric stress decreased with higher gel material content in comparison to stabilized specimens subjected to 100 kPa and 400 kPa confining pressures, regardless of gel material type, except for the G-stabilized specimen treated with 22% gypsum content.

As the $\sigma_{3}$ increases, the increase in the ${\left(\sigma_{1}-\sigma_{3}\right)}_{\max}$ of G-stabilized specimens with 22% gypsum gel material content is greater than that of G-stabilized specimens with 16% gypsum gel material content. This can be attributed to the unique mechanisms influencing the strength of stabilized calcareous sand. Upon mixing cement with calcareous sand and water, rapid hydration reactions occur. Initially, hydration products gradually fill surface pores on calcareous sand particles, and with higher gel material content, these products coat the particles. Ultimately, when the gel material content is sufficient, hydration products fill pores between the particles, enhancing interlocking and bonding, resulting in a stabilized specimen with a cohesive structure and improved load-bearing capacity. Further elaboration on this mechanism is available in another paper by the authors [11]. This leads to a higher ${\left(\sigma_{1}-\sigma_{3}\right)}_{\max}$ increase in the G-stabilized specimen with 22% gel material content compared to other gel material content levels as the confining pressure increases.

Notably, the peak deviatoric stress in stabilized specimens rose with escalating confining pressure, causing distinctive mechanical responses due to both the level of cementation and the breakage of calcareous sand particles. Illustrated in Figure 1a,c, and Figure 2a,c, post-yield strain-hardening responses were observed across diverse cementation levels (5% and 10% for Portland cement and 13% and 16% for gypsum), independent of the gel material type. Conversely, post-yield strain-softening responses were evident at higher cementation levels (16% for Portland cement and 22% for gypsum), as depicted in Figure 1e and Figure 2e. The interaction of breakage and cementation level prompted the shift from a stress–strain hardening to stress–strain softening response with increasing confining pressure $\sigma_{3}$. At low gel material content, the stabilized specimen initially withstands loading owing to structural interlocking and bonding strength. With increased loading, the specimen’s strength primarily stems from particle breakage, arrangement, and the inherent strength of sand particles. Additionally, a stress–strain hardening response is observed in all specimens, as depicted in Figure 1a, with comparable residual strength across respective confining pressures. This suggests that particle breakage governs the mechanical behavior of both tested specimens after reaching the ${\left(\sigma_{1}-\sigma_{3}\right)}_{\max}$ in the CU triaxial test. Moreover, it is evident that higher confining pressures lead to easier particle breakage, as reported by Coop [3] and Lyu et al. [38].

When the gel material content is adequate, the interlocking and bonding strength of stabilized specimens effectively supports incomplete loading. However, this has a restricted impact on improving the residual strength resulting from stress–strain softening, as illustrated in Figure 1e and Figure 2e. Residual strength is notably higher in stabilized specimens with adequate gel material content in comparison to those with lower gel material content and loose calcareous sand specimens. Moreover, the ${\left(\sigma_{1}-\sigma_{3}\right)}_{\max}$ of loose calcareous sand surpasses that of stabilized specimens. This disparity primarily stems from the added gel material content, determined by the weights of dry calcareous sand following guidelines provided by Suebsuk et al. [39]. This method maintained a consistent dry unit weight while increasing gel material content for each case, suggesting that lower gel material content results in a greater quantity of calcareous sand particles effectively resisting loading during shearing.

The shearing of stabilized specimens results in positive or negative pore water pressure, corresponding to compression or dilation during the triaxial shearing, as depicted in Figure 1 and Figure 2. Pronounced dilative changes in pore water pressure occur in stabilized specimens at larger axial strains and higher stress levels, especially near failure. Stabilized specimens displayed contraction at lower axial strain levels (ε_1_ ≤ 1~2%), followed by pronounced dilation. As the confining pressure increased, the contraction became more apparent, whereas the degree of dilation reduced. This suggests that heightened confining pressure leads to increased contraction due to cement bonding breakdown and particle breakage. Particle breakage notably contributes to substantial contraction at lower axial levels across various confining pressures, in contrast to the slight contraction observed at higher axial levels under different confining pressures. This observation demonstrates that stress–strain hardening correlates with a substantial strain at the yield point. As an illustration, the strains at the yield point were roughly 1.0% and 2.0% for 16% gypsum-stabilized specimens under 100 and 400 kPa confining pressures, as depicted in Figure 2c. This finding suggests that both the ${\left(\sigma_{1}-\sigma_{3}\right)}_{\max}$ and the associated strain level rose as the confining pressure increased [40,41]. The maximum negative pore water pressure in stabilized specimens decreased as the confining pressure increased after reaching the peak stress point. This reduction was linked to increased particle breakage of calcareous sand, which led to elevated pore water pressure, irrespective of gel material content. This suggests that under low confining pressure conditions, stabilized specimens were more likely to experience dilation during shearing.

**Figure 3:**
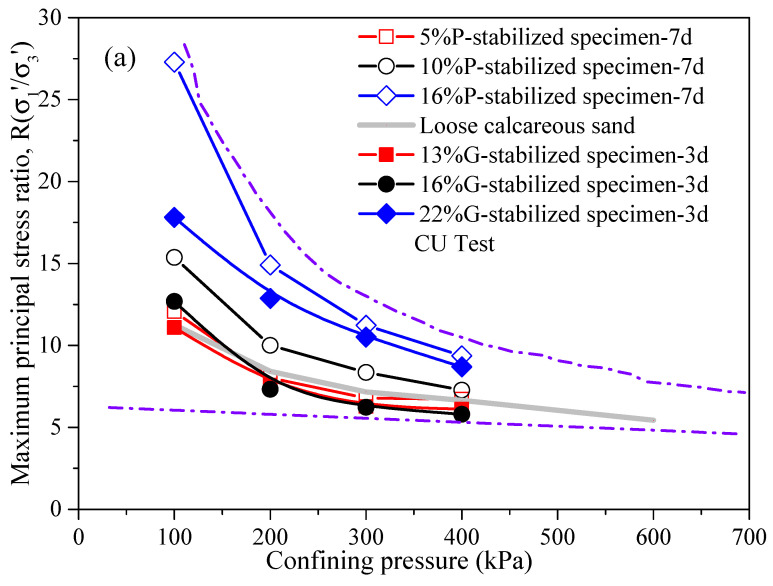

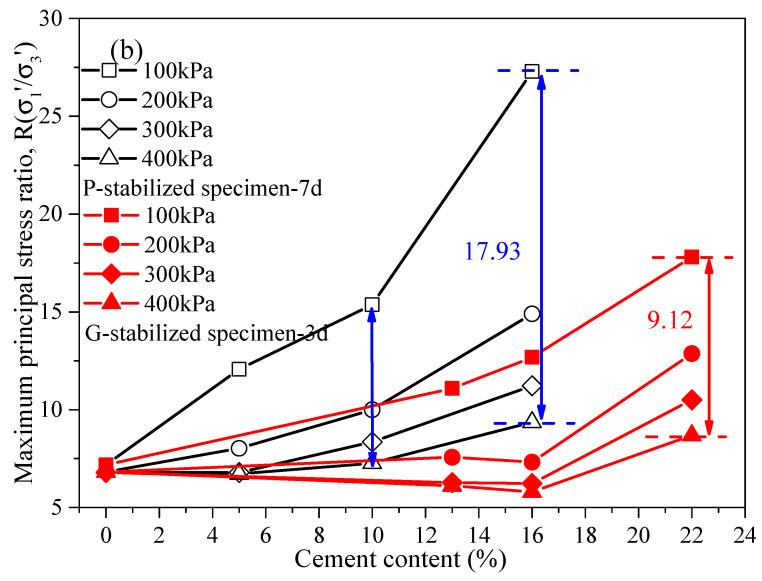
Maximum stress ratio versus confining pressure and cement content under different curing time conditions: (**a**) Effect of the confining pressure, and (**b**) effect of the cement content.

Figure 3a depicts the curves of principal stress ratios $R{\left({\sigma^{\prime }}_{1}\backslash {\sigma^{\prime }}_{3}\right)}_{\max}$ versus peak deviatoric stress. The value $R{\left({\sigma^{\prime }}_{1}\backslash {\sigma^{\prime }}_{3}\right)}_{\max}$ decreases with increasing confining pressure, regardless of the gel material type. Additionally, compared to the $R{\left({\sigma^{\prime }}_{1}\backslash {\sigma^{\prime }}_{3}\right)}_{\max}$ of loose calcareous sand, it stabilizes at a constant value under sufficiently high confining pressure, indicating a trend toward zero shear strength and bonding stiffness. Haeri and Hamidi [42] corroborated this inference in their experiment, and Gu et al. [11] offer an in-depth explanation. Hence, when employing cement to fortify the strength of calcium-based materials, the potential implications of higher-pressure settings, notably in deep-sea subjects, should be carefully evaluated.

### 2.2. Impact of Gel Material Content on Mechanical Behavior Response

**Figure 4:**
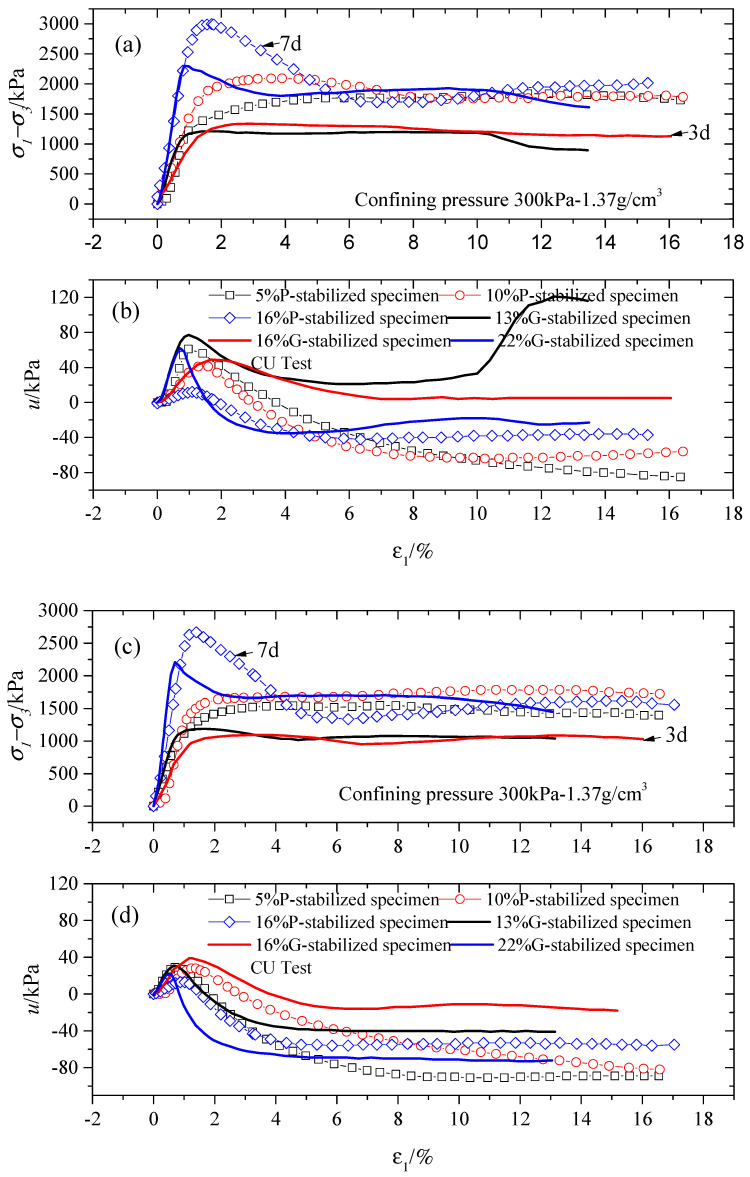

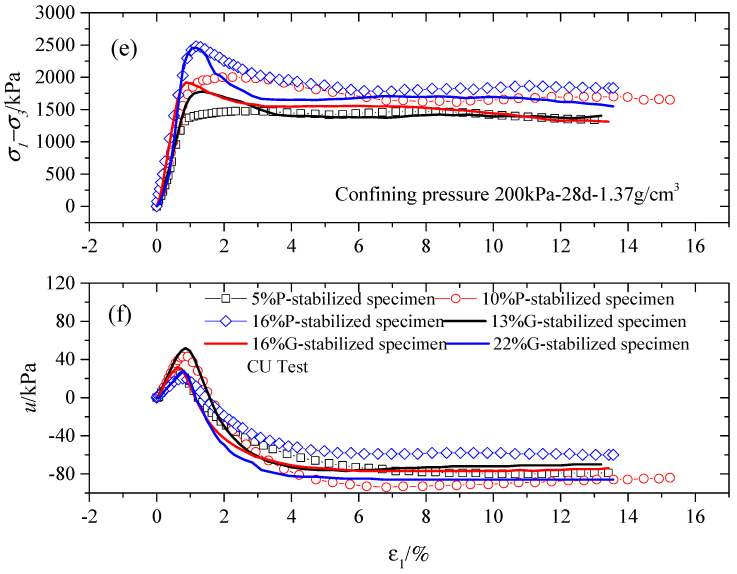
Results for specimens treated with different cement contents and cement types at 200 and 300 kPa confining pressure in the CU test. (**a**,**c**,**e**) Deviatoric stress versus axial strain; (**b**,**d**,**f**) pore water pressure versus axial strain.

Based on Figure 1, Figure 2, and Figure 4, it is evident that cementation has promoted the dilatancy of stabilized specimens in all tests. The increase in dilatancy increased subsequently, reaching its maximum after the peak stress point [43]. This suggests that the peak strength and dilatancy rarely coincide at the identical strain.

Figure 4 shows that the ${\left(\sigma_{1}-\sigma_{3}\right)}_{\max}$ of stabilized specimens rises with an increase in gypsum or Portland gel material content, independent of the gel material type. This indicates that cement notably increases the mechanical strength and rigidity of stabilized specimens via the impact of the interlocking and bonding structure [28,30,31,34,44]. Furthermore, unlike the findings reported by Sariosseiri and Muhunthan [34], all the stabilized specimens in the CU tests showed minimal signs of brittle breakage in their stress–strain curves. The stress–strain hardening behavior was noticed at low gel material content, regardless of the type of cement. However, the stress–strain response tended to soften after the yield point with an increase in gel material content, as shown in Figure 4a,c. This change primarily occurs because adequate hydration products coat and link sand particles, and consistently occupy or partition the pore volume. Consequently, this leads to a higher peak deviatoric stress, related to interlocking and bonding within the structure at the same experimental condition. The higher peak deviatoric stress contributes to the transition from a hardening response in the stress–strain curve to a softening response with increased gel material content. Ultimately, higher gel material content induces a more pronounced softening behavior in the results of the stress–strain curve.

As shown in Figure 3b, the $R{\left({\sigma^{\prime }}_{1}\backslash {\sigma^{\prime }}_{3}\right)}_{\max}$ increases with rising gel material content, regardless of the gel material type. For example, in the range of 100 kPa to 400 kPa confining pressure, the $R{\left({\sigma^{\prime }}_{1}\backslash {\sigma^{\prime }}_{3}\right)}_{\max}$ of a P-stabilized specimen with 16% Portland gel material content increased to roughly 2.26, 1.86, 1.65, and 1.39 times that of a P-stabilized specimen with 5% gypsum content at a 7-day curing period, respectively. In the case of the G-stabilized specimen, across the 100–400 kPa confining pressure range, the $R{\left({\sigma^{\prime }}_{1}\backslash {\sigma^{\prime }}_{3}\right)}_{\max}$ for the specimen with 22% gypsum content rose to approximately 1.61, 1.70, 1.67, and 1.42 times that of the G-stabilized specimen (13% gypsum content) for a 3-day curing period, respectively. Compared with the $R{\left({\sigma^{\prime }}_{1}\backslash {\sigma^{\prime }}_{3}\right)}_{\max}$ at confining pressures of 100 kPa and 400 kPa, it was observed that the value at 100 kPa was higher than that at 400 kPa, with increases of up to 17.93 and 9.12 for the 16% P-stabilized specimen and 22% G-stabilized specimen, respectively. Furthermore, when comparing the peak principal stress ratios of the 10% and 16% P-stabilized specimens at confining pressures of 100 kPa and 400 kPa, respectively, it was found that the increase decreased with increasing gel material content, irrespective of the gel material type.

### 2.3. Impact of Gel Material Type on Mechanical Behavior Response

Figure 4b,d,f demonstrate the occurrence of excessive negative pore water pressure after the point of yielding for stabilized specimens with increased gel material content and prolonged curing periods. This implies that the stabilized specimens were more likely to dilate during triaxial shearing due to the presence of numerous hydration products resulting from higher levels of cementation. Moreover, the P-stabilized specimens exhibited a higher tendency to dilate in the CU triaxial test under shorter curing periods, as illustrated in Figure 4b,d. It is observed that G-stabilized specimens exhibit a greater tendency for contraction during shearing compared to P-stabilized specimens. Ismail et al. [15,45] also reported a similar conclusion. The significant post-yield dilation observed in the P-stabilized specimens suggests a higher likelihood of the undrained shear strength exceeding the drained strength.

When comparing the P-stabilized and G-stabilized specimens in Figure 4a,c across various gel material contents, the peak deviatoric stress of the 7-day P-stabilized specimen increased to approximately 1.3 times that of the 3-day G-stabilized specimen, regardless of the gel material content. With the curing period increased to 28 days, the mechanical behavior of the G-stabilized specimens became similar to the P-stabilized specimens. Their peak deviatoric stress, residual strength, and peak pore water pressure displayed fluctuations, maintaining a constant value. A post-yield strain-softening response was observed in all stabilized specimens, depicted in Figure 4e.

### 2.4. Impact of Curing Period on Mechanical Behavior Response

**Figure 5:**
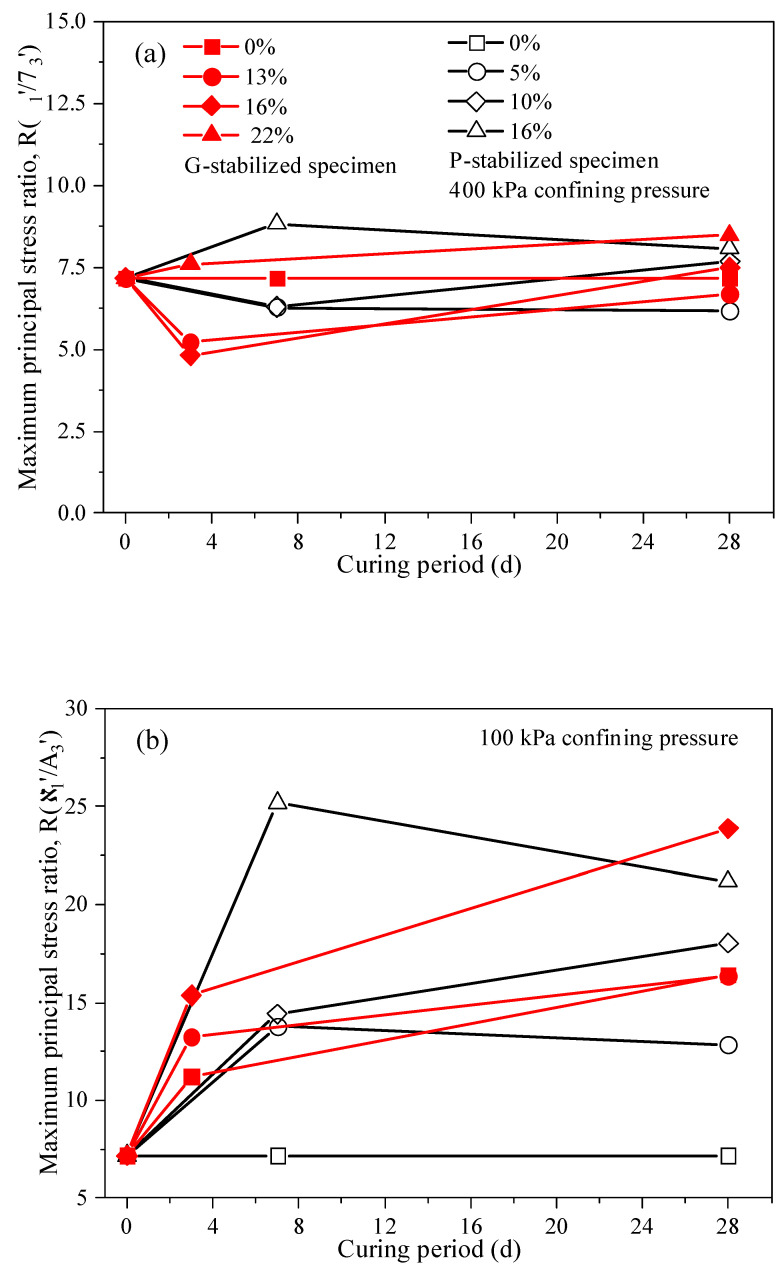
Results for maximum stress ratio versus curing period with different values of cement content. (**a**) Results of 400 kPa confining pressure; (**b**) Results of 100 kPa confining pressure.

Throughout all scenarios, the ${\left(\sigma_{1}-\sigma_{3}\right)}_{\max}$ of the stabilized specimens increased with prolonged curing periods. Table 1 displays the $R{\left({\sigma^{\prime }}_{1}\backslash {\sigma^{\prime }}_{3}\right)}_{\max}$ of stabilized specimens with 5–16% Portland gel material contents and 13–22% gypsum contents over varied curing durations (3 days, 7 days, and 28 days). Notably, at low confining pressure, the $R{\left({\sigma^{\prime }}_{1}\backslash {\sigma^{\prime }}_{3}\right)}_{\max}$ escalates with an extended curing period, depicted in Figure 5b. Conversely, under increased confining pressure, the parameter $R{\left({\sigma^{\prime }}_{1}{\sigma^{\prime }}_{3}\right)}_{\max}$ reaches a constant value, as shown in Figure 5a. This phenomenon, previously mentioned and explained as detailed in Section 2.1, suggests that the mechanical strength and rigidity of bonds diminish under sufficiently high confining pressures. It highlights how the mechanical response of the stabilized specimen is primarily influenced by particle strength, particle breakage, and their arrangement after reaching the peak deviatoric stress in the CU test.

### 2.5. Impact of Density on Mechanical Behavior Response

**Figure 6:**
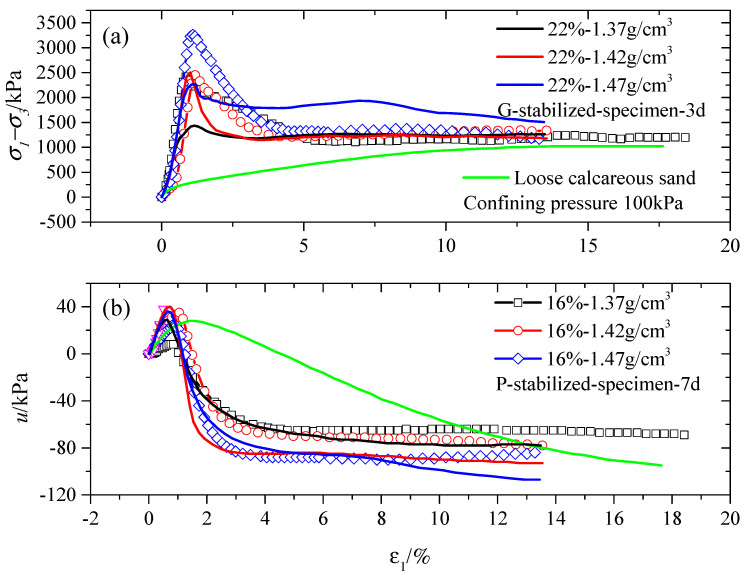
Results for specimens treated with 16% and 22% cement contents at 100 kPa confining pressure in the CU test. (**a**) Deviatoric stress versus axial strain, (**b**) pore water pressure versus axial strain.

Figure 6 illustrates the behavior of calcareous sand subjected to two different gel material types at three density levels (1.37, 1.42, and 1.47 g/cm^3^) for the elevated cementation condition. The outcomes reveal that the G-stabilized specimen exhibited the lowest strength increase (around 40% increase in peak deviatoric stress) compared to the 22%-1.37 g/cm^3^ G-stabilized specimen and loose calcareous sand (1.37 g/cm^3^), as shown in Figure 6b. Conversely, the P-stabilized specimens at 16%-1.37 g/cm^3^ displayed a more substantial strength increase of about 136%.

Moreover, the ${\left(\sigma_{1}-\sigma_{3}\right)}_{\max}$ of the stabilized specimens elevated with rising density, regardless of the gel material type. For instance, at a 100 kPa confining pressure, the ${\left(\sigma_{1}-\sigma_{3}\right)}_{\max}$ of the P-stabilized specimen with a density of 1.47 g/cm^3^ increased to approximately 1.35 times that of the P-stabilized specimen with a density of 1.37 g/cm^3^ for a 7-day curing period, respectively. Under the 100 kPa confining pressure, the ${\left(\sigma_{1}-\sigma_{3}\right)}_{\max}$ of the G-stabilized specimen with a density of 1.47 g/cm^3^ increased to approximately 1.58 times that of the G-stabilized specimen with a density of 1.37 g/cm^3^ for a 7-day curing period, respectively. It is evident that the impact of density on the G-stabilized specimen is more pronounced than that on the P-stabilized specimen, resulting in a higher peak deviatoric stress in the CU test.

### 2.6. Mechanical Properties of Stabilized Specimens

**Figure 7:**
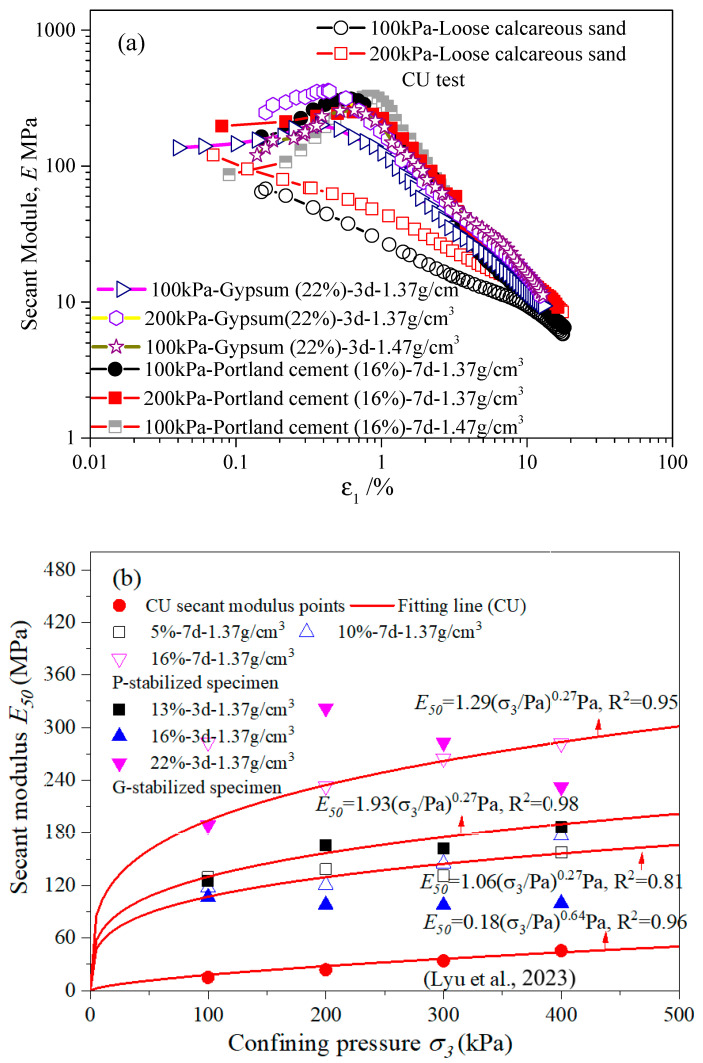
Strength parameters of stabilized specimens: (**a**) Stiffness modulus versus axial strain E–ε_1_ in a logarithmic scale for loose calcareous sand and stabilized specimen; and (**b**) secant modulus versus axial strain $E_{50}-\sigma_{3}$. Ref. [38] belongs to (**b**).

The strength parameters of stabilized specimens were graphed to examine the variation in stiffness during the shearing of treated specimens, as shown in Figure 7. It was noted that there is a distinct sharp bend in the results of all tests for stabilized specimens. Preceding this bend, the value of tangential stiffness maintains a nearly constant slope during triaxial shearing. Subsequently, it swiftly declines after this inflection point to match the behavior observed in untreated specimens, as depicted in Figure 7a. This juncture is typically referred to as the yield point of stabilized specimens, manifesting within a strain range of 0.5% to 5% in the CU test. The precise range is contingent on factors such as confining pressure, curing days, gel material type, and gel material content. Moreover, in Figure 7a it is evident that the stabilized specimen (3d-22%-1.47 g/cm^3^) reached the yield point at a lower axial strain under 100 kPa confining pressure compared to the stabilized specimen (7d-16%-1.47 g/cm^3^) under the same confining pressure. The parameter *E*_50_ represents the slope of the stabilized specimen at 50% of the peak strength (Table 1), offering insight into the average stiffness during triaxial shear.

.

As shown in Figure 7a, under shorter curing periods (7 days for P-stabilized and 3 days for G-stabilized specimens), the parameter *E* of the stabilized specimens increased with the effective confining pressure. With increasing gel material content, the secant modulus of the stabilized specimens notably improved, especially at higher gel material contents, regardless of the type of cement used. As depicted in Figure 7b, it also observed that the *E*_50_ values of stabilized specimens increased in a nonlinear fashion with increasing confining pressure. An example is the increase in the *E*_50_ value for the 10%-7d-1.37 g/cm^3^ stabilized specimen by 56.3 MPa when the confining pressure increased from 200 kPa to 400 kPa. Analogous findings were observed in the studies by Wang et al. [6] and Wu et al. [46]. It can be elucidated that the stabilized specimen developed a more compact structure, withstanding average deformation as the confining pressure increased, despite the presence of some imperfect data points.

Further analysis revealed that the correlation between *E*_50_ and the confining pressure can be formulated as below:

(1) $$E_{50}=a_{2}{\left(\frac{\sigma_{3}}{Pa}\right)}^{n}Pa$$

where *a*_2_ and *n* are adjustment parameters. *Pa* represents the value of one atmospheric pressure. The *E*_50_ value of the stabilized specimen ranged from 1 to 320, which is higher than that of loose calcareous sand (ranging from 1 to 110, as reported by Lyu et al. [38]). This variation depends on the intrinsic properties of calcareous sand, such as inner voids, mineral constituents, proportions, particle sizing, as well as factors like gel material content, gel material type, mean stress value, and curing period, as presented in Table 1 and Figure 7b. The influence of confining pressure on the *E*_50_ value was revealed through Equation (1). An example curve, as shown in Figure 7b, demonstrates that Equation (1) accurately describes the curves. In this way, the change in curves revealed the influence of confining pressure on the *E*_50_ value. In addition, the increase in the *E*_50_ value of the P-stabilized specimen was more significant than that of the G-stabilized specimen when compared with the value of loose calcareous sand in Figure 7b and Table 1. The strength of the specimens was significantly influenced by the Portland cement treatment compared to the gypsum-treated condition, leading to a more substantial resistance to loading.

**Figure 8:**
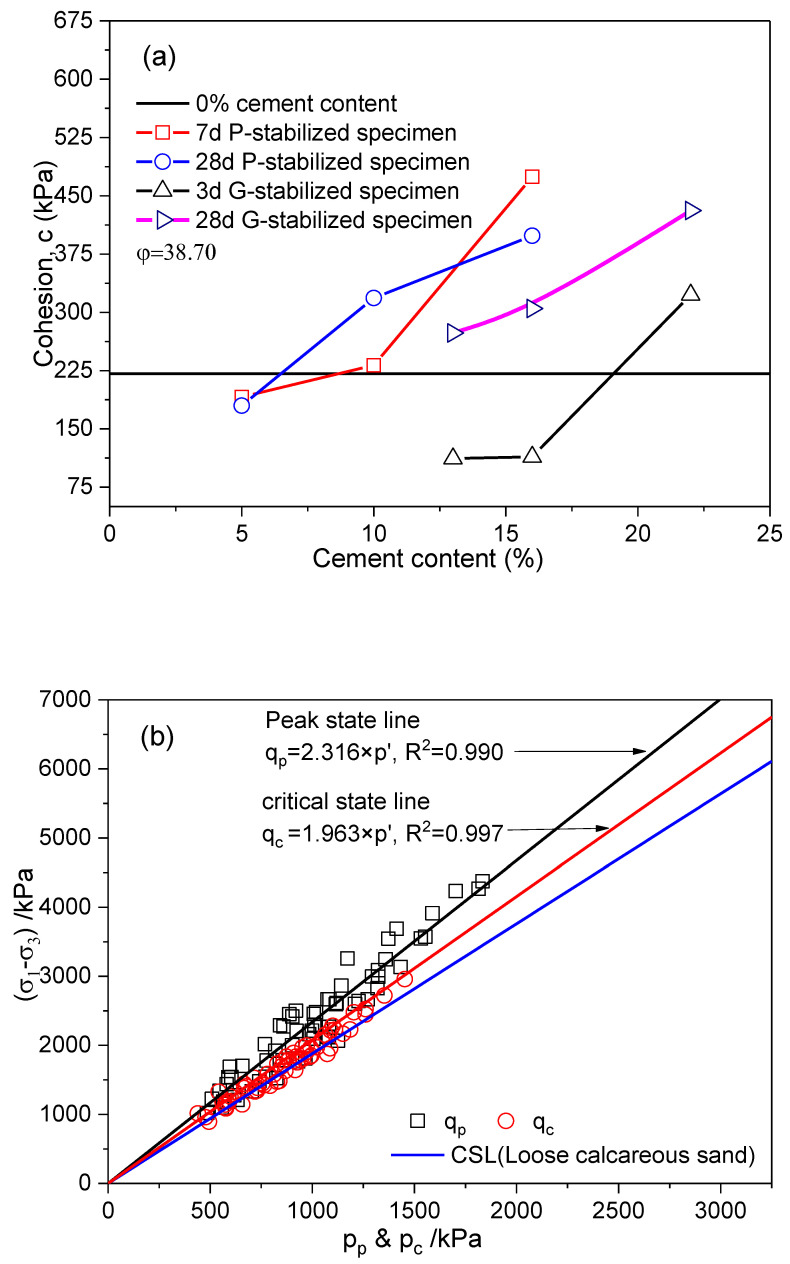
Strength parameters of stabilized specimens. (**a**) Cohesion, c, versus cement content: 0–22% cement content at 3–28 days curing; (**b**) peak strength, *q_p_*, and critical-state strength, *q_c_*, versus mean effective stress, $\sigma_{1}-\sigma_{3}$: 0–22% cement at 3–28 days curing.

The parameters M, friction angle φ, and cohesion c are calculated in Table 1. In this study, the failure point was defined as the peak deviatoric stress. The parameter φ characterizes the stabilized specimen’s ability to endure shear stress. It is considered to be approximately 38.7° for stabilized specimens due to the connection or coating of hydration products on the sand particles, which minimally altered the mineral composition (Gu et al. [11]). Table 1 and Figure 8a illustrate that the parameter c increased with higher gel material content and longer curing periods, aligning with the findings reported by Suebsuk et al. [39]. The increase in cohesion to a certain extent reflects the bonding strength of the stabilized specimen, reaching a maximum enhancement of up to 2.14 times. Moreover, the c values for the P-stabilized specimen with 5% Portland cement content at 7 days and 28 days of curing were lower than those of the untreated specimen. The c values of the 13%- and 16%-gypsum-content stabilized specimens after a 3d curing period were lower than that of the specimen without treatment. It was observed that there is a minimum requirement for cement agent content to improve the strength of stabilized calcareous sand.

Moreover, the parameter c of the stabilized specimens between 110 and 475 kPa with cement exhibited a substantial enhancement compared to the values between 16 and 308.5 kPa in a specimen without treatment [47,48]. It was known that the mechanical response and strength characteristics of the specimen were significantly reinforced. Figure 8b categorized all peak and critical state points according to the mean effective stresses. The critical state point is defined as the axial strain of 13%, at which the stabilized specimen does not exhibit an obviously critical state response. Figure 8b displays three distinct lines, representing all triaxial tests conducted at different confining pressures. The parameter M has values of 2.31, 1.96, and 1.88 for the peak state, the critical state of the stabilized specimen, and the critical state of calcareous sand, respectively. It is worth noting that the factors of cement agent content and curing time seemed to impact the critical state line. Two suppositions were formulated concerning the increase in the value of the parameter M. The first suggests that particles might remain bonded (bonding effect), potentially enhancing the strength of the stabilized specimen. The second point suggests that various sizes of cemented clusters persisted at this critical state, forming more resilient force-chain networks to enhance the residual strength, as elucidated by Gu et al. [11].

The brittleness of stabilized specimens relies on the cement agent content, curing time, and gel material type. The parameter I_B_ (=q_peak_/q_resid_ − 1, brittleness index) has been defined to depict the brittleness of specimens, in which q_peak_ is the peak deviatoric stress, and q_resid_ is the residual strength. Table 1 shows the parameter I_B_ results calculated from the CU tests of stabilized specimens. The parameter I_B_ increased with increasing gel material contents or curing periods, irrespective of the gel material type. In addition, the brittleness index I_B_ decreased with increasing confining pressure.

## 3. Conclusions

This paper investigated the impact of gel material content, curing period, confining pressure, gel material type, and density on the CU test and UCS test of stabilized specimens. Various tests were conducted, altering the content of Portland cement (5%, 10%, and 16%), gypsum cement (13%, 16%, and 22%), density (1.37 g/cm^3^, 1.42 g/cm^3^, 1.47 g/cm^3^), and curing period (3 days, 7 days, and 28 days). The mechanical behavior of the stabilized specimens was assessed concerning gel material content, curing period, confining pressure, gel material type, and density. The evolution of peak deviatoric stress from the CU test revealed the impact of gel material content, gel material type, and curing period on strength. The strength parameters discussed include the principal stress ratio, secant modulus, cohesion, friction angle, and brittleness index. The study’s results encompass the following specific details:

1. The findings from the UCS test revealed a positive correlation between q_ucs_ and the increasing gel material content. Three cementation levels were chosen to explore the impact of gel material type, characterized by cement/sand ratios of 5%, 10%, and 16% for Portland cement and 13%, 16%, and 22% for gypsum.
2. CU tests demonstrated a substantial strength increase with rising curing periods or confining pressure. With increased curing periods or confining pressure, noticeable strain hardening and dilation were observed after the post-yield point in the results. At high gel material contents, an excessive negative pore water pressure was generated in the stabilized specimens beyond the post-yield point, resulting in observed strain softening. This phenomenon suggests an increased propensity for dilation with rising gel material content, irrespective of the gel material type.
3. The peak principal stress ratio, strength parameters, secant modulus, and cohesion increased with increasing curing period and gel material content. In addition, the relationship between the secant modulus *E*_50_ and confining pressure was qualitatively explained. The study illuminates the impact of key factors on cement-treated calcareous sand, providing insights into stabilized specimen behavior and contributing to the development of more efficient and cost-effective methods for calcareous sand improvement.

The mechanical behavior and cement quantity of treated calcareous sand in this study sheds light on the behavior of stabilized specimens and helps to develop more efficient and cost-effective methods of calcareous sand improvement.

## 4. Materials and Methods

### 4.1. Materials

#### 4.1.1. Calcareous Sand and Portland Cement

**Figure 9:**
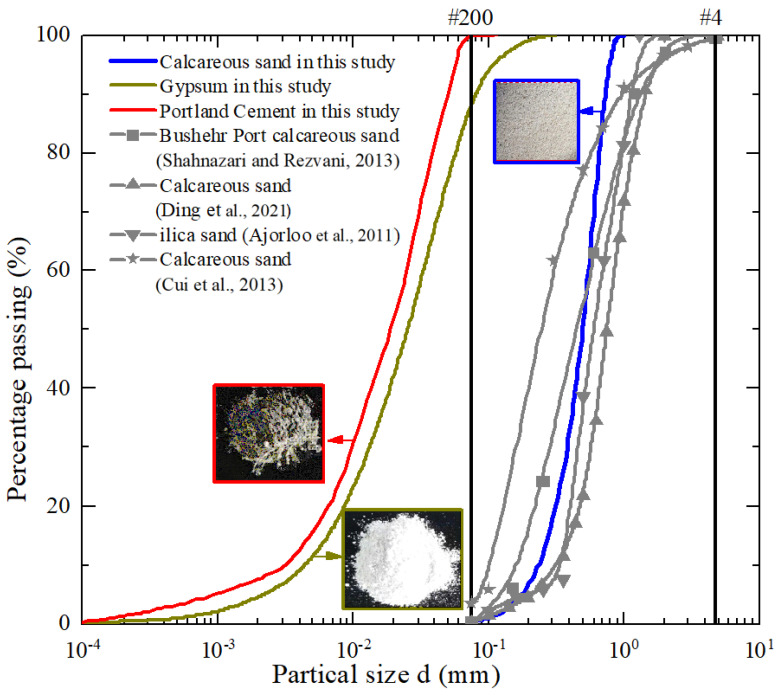
Grain size distribution curves for test materials [43,49,50,51].

Calcareous sand from Sansha, Hainan province, China, known for its leeward and low-wave coastal characteristics near Sanya City, was carefully prepared to ensure suitability for the experimental investigation. Comprised mainly of low-strength fossils and shells, the calcareous sand displayed unique compressible and brittle traits. The sand was soaked in distilled water for three days, with water changes three times daily to remove soluble salt and any residues. Subsequently, it underwent heat sterilization at 100 °C for 24 h and was sieved through a 1 mm sieve before experimentation. Microscopic analysis by Shahnazari and Rezvani [49] identified irregular grain shapes and abundant pores in the calcareous sand, with prevalent components such as thin-walled mollusks and echinoderm plate fragments observed through a scanning electron microscope (SEM). The calcareous sand, detailed in Table 2, is primarily composed of calcium carbonate, with a significant 91.75% calcium content, determined via precise EDTA complexometry testing, marking it as a unique geomaterial with a CaCO_3_ content surpassing 50% (Gu et al. [11]). Figure 9 depicts the grain size distribution of the specimen, revealing a lack of fines, which indicates that the selected sands were well-graded sand. The Portland cement used in this study is consistent with the authors’ previous research (Gu et al. [11]). Additionally, its specific gravity was measured at 3.1, and it had a CaO mineral component ranging from 62% to 67% (Table 2).

#### 4.1.2. Gypsum

Gypsum, among various cementitious materials, presents a relatively inexpensive binding option compared to Portland cement. Its presence offers significant potential for widespread use in materials that improve the strength of foundation bases. Additionally, it can serve as a comparative binder to examine the impact of gel material type on the mechanical behavior of cemented calcareous sand. The selected gypsum is β-type semi-hydrated gypsum, commonly available in the form of construction-grade gypsum powder. Its average size (D_50_) measures 0.024 mm, and the specific gravity of gypsum before hydration is approximately 2.4.

Gypsum demonstrates a setting time with an onset at 6 min and completion at 15 min. The water content employed to determine the setting time aligns with the 10% water content specified in the sample preparation. However, this setting time does not meet the sample preparation time requirements for gypsum-cemented calcareous sand, potentially leading to strength discrepancies among different specimens. To meet the sample preparation time requirements for gypsum-cemented calcareous sand, a new commercial retarding agent, CQ-SHJ09, was identified. When applied at a proportion of 0.4% relative to the gypsum mass, it effectively extends the average initial setting time of gypsum to approximately 50 min while maintaining the specimen’s water content at around 10% during setting testing.

Moreover, the gypsum retarding agent is a novel, high-performance retarding agent based on proteins. Its primary advantages involve a reduced dosage compared to traditional retarding agents at the same content, leading to an extended retarding time. The retarding effect is noteworthy, significantly minimizing the strength loss in gypsum, nearly preserving the strength of gypsum-cemented calcareous sand specimens.

### 4.2. Sample Preparation

The amounts of Portland cement and gypsum added were computed using the dry weight of the sand, following the specifications of Suebsuk et al. [39]. This approach ensured a uniform dry unit weight of stabilized specimens while adjusting the gel material content for each particular case. At the outset, Portland cement or gypsum, dry calcareous sand, and water at the optimal moisture content were carefully combined using the dry-mixing technique [3] until a uniform mixture was achieved. This involved weighing the calcareous sand and cement, followed by thorough mixing in a bowl until a consistent and uniform blend was obtained. Subsequently, 10% distilled water was added to the mixture in proportion to the target specimen weight, mixed until a consistent paste was attained. The selection of a 10% distilled water content was grounded in the authors’ previous experience, in which it demonstrated its effectiveness in facilitating the sampling process.

A cylindrical split mold (diameter: 39.10 mm, height: 80.00 mm) was used to compress the stabilized specimens into four equal layers, to attain the desired specimen density. The sampling duration for the stabilized specimens has a significant impact on the ultimate shear strength [52]. To avoid premature solidification arising from the interaction of chemical reinforcement, all sampling procedures were meticulously carried out within a 50 min timeframe. This measure was taken to preserve the integrity of the specimens and ensure their suitability for subsequent testing and analysis in this study. Following molding, the stabilized specimens underwent an immediate vacuum saturation test and were then cured in distilled water at a controlled temperature of 20 °C ± 2 °C. This approach was employed to alleviate the influence of capillary action on specimen strength, as highlighted by Consoil et al. [53], thereby ensuring the precision of strength measurements.

Subsequent to the curing time, stabilized specimens were removed from the curing room, and only those meeting the specified tolerances were retained. These tolerances encompassed densities of 1.37 g/cm^3^, 1.42 g/cm^3^, and 1.47 g/cm^3^, with a tolerance of ±0.01 g/cm^3^. Additionally, the dimensions were scrutinized, permitting for a variation of ±0.5 mm in diameter (39.10 mm) and ±1 mm in height (80.00 mm). These stringent selection criteria were enforced to ensure the precision and dependability of the test outcomes. The preparation steps of loose calcareous sand are introduced in detail by Lyu et al. [38] and Wang et al. [27].

### 4.3. Comparative Experimental Design

Investigating the influence of gel material type on stabilized specimen properties necessitates consistent sample preparation. Achieving consistency involves maintaining uniform density and unconfined compressive strength across similar samples, as noted by Ismail et al. [15]. A parametric study was carried out to establish the relationship between unconfined compressive strength (q_ucs_) and cement percentage. The typical range for gel material content in stabilized soil foundations is 5% to 25% based on soil weight [34,54,55]. It is vital to control the gel material content within a specified range to manage increased self-weight stress induced by reinforcement materials in composite foundations. Additionally, maintaining soil strength while reducing self-weight is essential. In this research, the stabilized calcareous sand had cement percentages ranging between 5% and 24%, ensuring optimal proportions of Portland cement and gypsum for the consolidation triaxial test.

**Figure 10:**
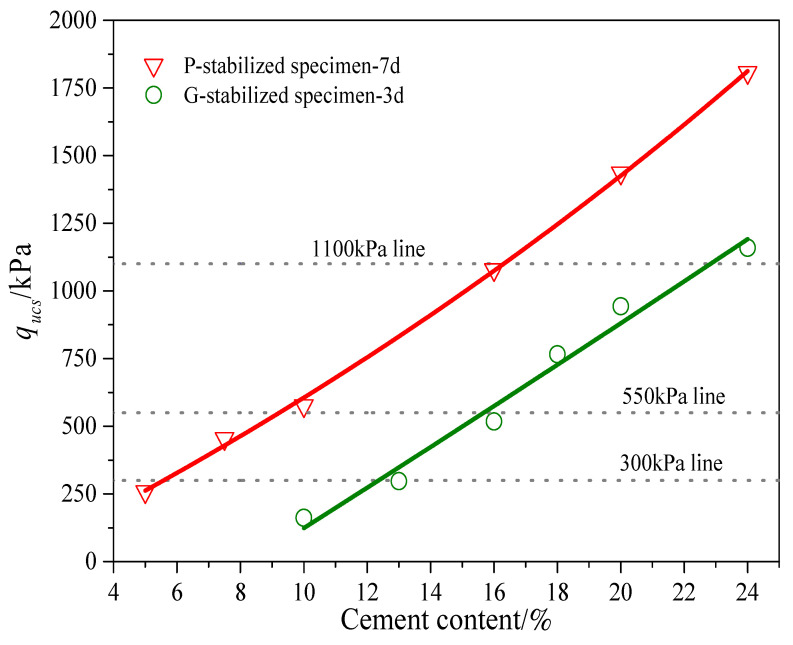
Relationship between *q*_ucs_ and cement content for stabilized specimens. Three levels of cementation were chosen for further study: *q*_ucs_ values of approximately 300 kPa, 550 kPa, and 1100 kPa. The corresponding cement/sand ratios to achieve these values were 5%, 10%, and 16% for Portland cement and 13%, 16%, and 22% for gypsum, respectively.

The experimental program for the UCS tests is detailed in Table 3. Notably, six sets of P-stabilized specimens using Portland cement were prepared with varying gel material content, 5%, 7.5%, 10%, 16%, 20%, and 24%, followed by a 7-day curing period. Additionally, six sets of gypsum-stabilized specimens (G-stabilized specimens) with gel material contents of 10%, 12%, 16%, 18%, 20%, and 24% underwent a 3-day curing period. The standard dry density was recorded as 1.37 g/cm^3^. The relationship between q_ucs_ and cement agent content is shown in Figure 10 for both the G-Stabilized and P-Stabilized specimens. The decision was made to choose three levels of cementation for further investigation, with the first one at q_ucs_ = 300 kPa, the second one at about 550 kPa, and the third one at about 1100 kPa. The cement/sand ratios corresponding to achieving these values of q_ucs_ were 5%, 10%, and 16% for Portland cement, and 13%, 16%, and 22% for gypsum, respectively. The cement/soil ratio mentioned here corresponds to the weight of the cement relative to the total dry weight of the stabilized specimens.

### 4.4. UCS Test and Triaxial Test Design

The UCS test is commonly employed to assess how various factors affect the strength of soils treated with cement. Its popularity stems from its ease of use and cost effectiveness. Prior to the UCS test, measurements of weight, height, and diameter were recorded. Detailed information on the UCS test process is provided in another study by the authors [44], and the experimental program, outlined in Table 3, enables the analysis of the impact of gel material content.

Triaxial tests are conducted to investigate the impact of cement agent content, confining pressure, and density on the mechanical behavior of stabilized specimens under consolidated undrained conditions, utilizing a strain-controlled triaxial test apparatus for both loose calcareous sand and stabilized specimens. Additionally, the B-value test was performed to ascertain the stabilized specimens were completely saturated. Initially, a confining pressure of 20 kPa was applied to the stabilized specimens, followed by a back pressure of 200 kPa to achieve a test value up to 0.95. Throughout this test, a mean effective confining pressure of 20 kPa was consistently upheld. The strain rate was set at 0.2 mm/min to eliminate any potential creep effects [15,45,56]. For the loose calcareous sand, the test process of the triaxial test was previously described thoroughly by the authors (Lyu et al., [38]). Table 4 shows the experimental program of triaxial undrained tests.

The study employed two sets of saturated stabilized specimens: one set contained Portland cement with varying gel material content (CC = 5%, 10%, and 16%), while the other set comprised gypsum with CC values of 13%, 16%, and 22%. Each set comprised four specimens undergoing distinct curing periods. These specimens were utilized for undrained triaxial shear tests to consolidate at varying net confining pressures (100 kPa, 200 kPa, 300 kPa, and 400 kPa). Moreover, a specific set of saturated stabilized specimens with 16% gel material content for Portland cement and 22% for gypsum was used to explore the density effect under identical net confining pressures.

## Figures and Tables

**Table 1:** Strength parameters of stabilized specimens: 0–16% cement and 0.1–0.4 MPa confining pressure, at 3–28 days curing (*ρ_d_* = 1.37 g/cm^3^, secant modulus, *E*_50_, *R = (σ*_1_*/σ*_3_*)*_Max_, *M*, *c*, and correlation coefficient *R*^2^, CU test).

Type			0% Cement Content	5% Cement Content		10% Cement Content		16% Cement Content		13% Cement Content		16% Cement Content		22% Cement Content	
				P-Stabilized Specimen						G-Stabilized Specimen					
Curing Period			0	7	28	7	28	7	28	3	28	3	28	3	28
Confining pressure (σ_3_ = MPa)	0.1	*E*_50_ (×10 MPa)	1.51	12.95	17.22	11.73	24.37	28.32	30.18	12.50	16.05	10.69	20.22	18.86	21.78
	0.2		2.39	13.85	16.79	12.06	20.78	23.32	27.60	16.57	17.97	9.80	24.84	32.22	20.45
	0.3		3.39	13.11	18.12	14.51	19.39	26.49	26.46	16.20	21.21	9.74	25.22	28.29	23.76
	0.4		4.56	15.76	22.38	17.69	22.12	28.19	32.04	18.63	22.55	9.98	22.62	23.23	25.30
	0.1	*R (σ*_1_ */σ*_3_ *)*_Max_	11.26	13.77	12.87	14.42	18.03	25.19	21.17	11.21	16.42	13.26	16.36	15.36	23.89
	0.2		8.41	8.72	8.4	9.92	11.01	14.33	13.41	6.93	9.89	6.48	10.6	12.04	13.29
	0.3		7.15	6.89	7.04	7.98	8.55	10.99	9.65	5.03	8.21	5.45	8.38	8.67	9.71
	0.4		6.65	6.28	6.16	6.3	7.67	8.84	8.07	5.22	6.68	4.81	7.50	7.60	8.49
	0.1	*I_B_*	0	0.26	0.34	0.12	0.32	0.99	0.39	0.04	0.59	0.23	0.35	0.14	0.67
	0.2		0	0.07	0.11	0.04	0.21	0.65	0.35	0.14	0.27	0.03	0.46	0.52	0.59
	0.3		0	0.06	0.12	0.17	0.17	0.49	0.31	0.35	0.58	0.18	0.25	0.43	0.28
	0.4		0	0.12	0.12	0.04	0.17	0.28	0.12	0.14	0.24	0.06	0.47	0.40	0.32
*M*			1.88	1.96	1.9	2.08	2.2	2.35	2.32	1.99	2.22	1.97	2.25	2.27	2.39
*R* ^2^ *_M*			0.992	0.980	0.946	0.859	0.834	0.492	0.644	0.724	0.822	0.358	0.891	0.940	0.592
*C* (kPa)			221.1 (φ = 38.7°)	191.12	179.91	231.89	318.43	474.64	398.62	111.91	273.45	114.01	304.82	322.67	430.88
*R* ^2^ *_c*			0.992	0.997	1	0.978	0.997	0.995	0.982	0.975	0.993	0.965	0.999	0.966	0.986

**Table 2:** Basic properties of calcareous sand and major mineral components of Portland cement.

Parameters		Value
Calcareous sand	*G*_s_	2.73
	*e*_max_	1.29
	*e*_min_	0.70
	CaCO_3_/%	91.75
	*C*_c_	0.95
	*C*_u_	2.07
	*d*_10_/mm	0.3
	*d*_60_/mm	0.62
	*d*_30_/mm	0.42
Portland cement components	CaO	62.0–67.0%
	SiO_2_	20.0–24%
	Al_2_O_3_	4.0–7.0%
	Fe_2_O_3_	2.5–6.0%

**Table 3:** Experimental program of unconfined compressive strength tests (UCS tests).

Test Type	Specimen	Cement Content (%)	Curing Period (d)	Density (g/cm^3^)	Initial Water Content (%)	Total Specimens
UCS Test	P-stabilized specimen	5, 7.5, 10, 16, 20, 24	7	1.37 (±0.01)	10 (±0.4)	3 × 6
	G-stabilized specimen	10, 12, 16, 18, 20, 24	3			3 × 6

**Table 4:** Experimental program of triaxial tests.

Type	Test	Cement Content (%)	Curing Period (d)	Confining Pressure (kPa)	Density (g/cm^3^)	Initial Water Content (%)	Total Specimens
Calcareous sand	CU	0	0	100, 200, 300, 400	1.37 (±0.01)	10 (±0.4)	4
P-stabilized specimen		16	7		1.42 (±0.01)		4
					1.47 (±0.01)		4
		5, 10, 16	7, 28		1.37 (±0.01)		24
G-stabilized specimen		22	3		1.42 (±0.01)		4
					1.47 (±0.01)		4
		13, 16, 22	3, 28		1.37 (±0.01)		24

## Data Availability

The original contributions presented in the study are included in the article, further inquiries can be directed to the corresponding author.
